# Multiclass Skin Lesion Classification Using Hybrid Deep Features Selection and Extreme Learning Machine

**DOI:** 10.3390/s22030799

**Published:** 2022-01-21

**Authors:** Farhat Afza, Muhammad Sharif, Muhammad Attique Khan, Usman Tariq, Hwan-Seung Yong, Jaehyuk Cha

**Affiliations:** 1 Department of Computer Science, Wah Campus, COMSATS University Islamabad, Wah Cantt 47040, Pakistan; farhatafza_std@ciitwah.edu.pk; 2 Department of Computer Science, HITEC University Taxila, Taxila 47080, Pakistan; attique.khan@hitecuni.edu.pk; 3 College of Computer Engineering and Science, Prince Sattam Bin Abdulaziz University, Al-Kharaj 11942, Saudi Arabia; u.tariq@psau.edu.sa; 4 Department of Computer Science & Engineering, Ewha Womans University, Seoul 03760, Korea; hsyong@ewha.ac.kr; 5 Department of Computer Science, Hanyang University, Seoul 04763, Korea; chajh@hanyang.ac.kr

**Keywords:** skin cancer, contrast enhancement, deep learning, evolutionary algorithms, fusion, ELM

## Abstract

The variation in skin textures and injuries, as well as the detection and classification of skin cancer, is a difficult task. Manually detecting skin lesions from dermoscopy images is a difficult and time-consuming process. Recent advancements in the domains of the internet of things (IoT) and artificial intelligence for medical applications demonstrated improvements in both accuracy and computational time. In this paper, a new method for multiclass skin lesion classification using best deep learning feature fusion and an extreme learning machine is proposed. The proposed method includes five primary steps: image acquisition and contrast enhancement; deep learning feature extraction using transfer learning; best feature selection using hybrid whale optimization and entropy-mutual information (EMI) approach; fusion of selected features using a modified canonical correlation based approach; and, finally, extreme learning machine based classification. The feature selection step improves the system’s computational efficiency and accuracy. The experiment is carried out on two publicly available datasets, HAM10000 and ISIC2018. The achieved accuracy on both datasets is 93.40 and 94.36 percent. When compared to state-of-the-art (SOTA) techniques, the proposed method’s accuracy is improved. Furthermore, the proposed method is computationally efficient.

## 1. Introduction

Skin cancer is caused by the abnormal growth of skin cells. Skin cancer cases have increased dramatically in recent years [1]. The increase in skin cancer is because of the reduction in the ozone layer, which is protection against ultraviolet rays [2]. Squamous cell carcinoma, actinic keratosis (solar keratosis), melanoma, and basal cell carcinoma are all types of skin cancer [3]. Melanoma is the most dangerous type of skin cancer, out of all the others. Melanoma begins in the melanocyte cells, which resemble a mole and are brown or black in color [4]. The occurrence rate of melanoma is 7%, but 75% of deaths are caused by this type of deadly cancer [5].

In 2017, in the USA, 3590 deaths occurred from 95,360 cases. From these cases, 87,110 were melanoma cases. The reported deaths in 2018 are 13,460 from a total of 99,550 cases. The melanoma cases that occurred in 2018 are 91,270. In 2019, 104,350 cases of skin cancer were reported in the USA alone. The number of reported cases in men was 62,320 and in women was 42,030. The number of melanoma cases was 96,480 (reportedly 57,220 in men and 39,260 in women) of all skin cancer cases reported in 2019. The number of deaths in 2019 due to melanoma was 7320 [6]. More than 15,000 people in the USA die every year from skin cancer lesions. In the next few decades, the mortality rate by melanoma infections could increase [7].

Because of the differences in skin textures and injuries, detecting skin cancer is a difficult task. As a result, dermatologists employ a noninvasive technique known as dermoscopy to detect skin lesions at an early stage [8]. The first step in dermoscopy is to apply the gel to the infected area. Then, a magnified image is acquired by using a magnifying tool. This magnified image provides a better visualization to examine the structure of the lesion area. The detection accuracy depends on the experience of the dermatologist [9]. A study shows that the dermatologist’s detection accuracy can vary between 75% and 84% [10]. Manual identification of skin lesions using dermoscopy, on the other hand, is a time-consuming procedure with a high risk of error, even for experienced dermatologists [11]. Therefore, researchers have introduced different computer-aided diagnostic (CAD) techniques based on machine learning and deep CNN features [12].

Dermatologists can use CAD systems to identify skin lesions more quickly and accurately. A CAD system’s key steps are skin image dataset acquisition, feature extraction and selection, and classification [13]. The utilization of deep features for skin lesion detection and classification has been shown to be of immense importance in the last few years compared to the traditional feature extraction techniques [14]. The deep features are extracted from the fully connected layers of a CNN model that was later employed for the classification [15]. Deep features, as opposed to traditional methods, such as texture, color, and shape, include both local and global information about an image. An image’s local information is extracted from the convolutional layer, while the global information is captured from the 1D layers (global average pooling and fully connected) [16]. In the traditional methods, shape features, such as HOG, color, and texture (LBP), are extracted separately [17].

A few researchers faced the problem of redundant features that mislead the multiclass lesion classification. Therefore, it is essential to develop a feature selection technique that selects the most appropriate features for final classification [18]. A few recently introduced feature selection techniques include grasshopper, binary whale optimization, among others [19]. As compared to the binary class classification problem, the multiclass classification is more complex due to the similar nature of skin lesions. Moreover, the intraclass similarity is another problem for the multiclass classification.

In this work, a new framework is proposed based on the hybrid whale optimization deep feature selection that later fused with a modified canonical correlation approach. The contrast stretching method is proposed to improve the visibility quality of the lesion region. The deep learning model is trained on the contrast enhanced lesions instead of original dermoscopic images. Further, two feature selection techniques are utilized in order to improve the accuracy and reduce the computational time.

## 2. Related Work

Several techniques and methods have been developed for the robust segmentation and classification of skin lesions [8]. The developed techniques utilized computer vision-based machine learning techniques [20]. Machine learning algorithms utilized supervised learning and deep learning methods for robust detection, segmentation, and classification of skin lesions [21]. Dorj et al. [22] utilized the deep features from pre-trained AlexNet model and that had been given to SVM for classification. Their technique produced promising results for lesion classification. Ren et al. [23] presented a fusion mechanism for the segmentation of a skin lesion. The spatial attention and channel attention modules extracted the information from channels of skin images. The implementation of serial network fusion for segmentation achieved competitive accuracy. Automated skin lesion segmentation [24] and classification was performed using the deep CNN-based mutual boot strapping network. The segmentation and classification used learned information of both phases using a bootstrapping technique. The proposed method was implemented on challenging skin lesion datasets and achieved robust segmentation and classification performance. A deep learning [25] technique was deployed to detect and classify the melanoma from dermoscopic images. A deep residual network was used to extract deep feature and fisher vectors utilized for image encoding. SVM classification was performed with chi-square to detect and classify the melanoma from discriminatory images of dermoscopy. The presented method achieved robust performance on the challenging ISBI 2016 dataset. Khan et al. [26] came up with a novel deep learning-based methodology for efficient segmentation and classification of skin lesions. The segmentation was performed using mask recurrent neural network (Mask-RNN). A pyramid network feature was used with Resnet50 for feature extraction and lesion classification performed with the SoftMax classifier. The HAM10000 dataset was utilized for the presented deep learning method evaluation and achieved competitive performance. A deep learning-based novel cascaded architecture [27] presented the ability for recognition of skin lesions. The diffusion of knowledge was required to perform analysis of the skin lesion and segmentation of lesions. The data augmentation was performed to remove the class disparity and implanted technique compared with the state-of-the-art (SOTA) technique of skin lesion classification and segmentation. The super pixel [28] method adopted for efficient segmentation and recognition of skin lesions used a novel segmentation technique. Image registration and segmentation techniques were fused for feature derivation, in order to achieve the better lesion segmentation results. The deep learning-based techniques [29] achieve robust performance in skin lesion segmentation, detection, and classification. Sikkandar et al. [30] came up with a new GrabCut and neuro fuzzy algorithm for recognition and segmentation of skin lesion. Preprocessing performed using top hat filter, GrabCut method used for segmentation, feature extraction performed using deep CNN model inception, and images of skin lesions classified using neuro fuzzy classifier. The presented technique applied on ISIC dataset and achieved robust performance in terms lesion segmentation and classification. Researchers in [31] used the transfer learning technique on the PH2 dataset for the classification of skin lesions. They performed transfer learning on the AlexNet network and achieved an accuracy of 98.61%.

The methods described above follow some standard steps for lesion classification, including preprocessing of lesion images, segmentation of skin lesions, extraction of deep learning features from segmented lesions, and, finally, classification using supervised learning methods. The accuracy of features extracted using pre-trained deep learning models is higher than that of features extracted using traditional techniques. The key limitation of the methods described above is the inclusion of redundant features, which not only reduces system accuracy but also increases computational time. Furthermore, the majority concentrated on the binary class classification problem. In this paper, we proposed a new framework for multiclass classification using deep learning and fusion of best selected features.

## 3. Datasets

Two datasets are utilized in this work for the experimental process, such as HAM10000 and ISIC2018. The detail of both datasets is given below.

**Figure 1:**
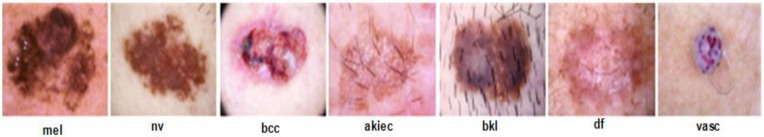
Sample images of HAM1000 dataset. melanoma (mel), melanocytic nevus (nv), basal cell carcinoma (bcc), actinic keratosis intraepithelial carcinoma (akiec), benign keratosis (bkl), dermatofibroma (df), and vascular lesion (vasc).

**HAM1000 Dataset:** The HAM10000 “Human Against Machine with 10,000 training images” dataset is one of the largest datasets, which contains 10,015 total dermoscopy images, used for detecting pigmented skin lesions, that are publicly accessible through the ISIC repository [32]. This dataset is grouped into seven different classes with a number of images, i.e., melanocytic nevus (nv = 6705), actinic keratosis (akiec = 327), dermatofibroma (df = 115), basal cell carcinoma (bcc = 514), vascular lesion (vacs = 115), benign keratosis (bkl = 1099), and melanoma (mel = 1113) [33]. The dataset contains 54% male and 45% female skin lesion images. It is a complex dataset with many skin lesion images having low inter-class and high intra-class variation issues, therefore the classification of these skin classes is not an easy task, and the chances of a high misclassification rate are significant. A few sample images are shown in Figure 1.

**Figure 2:**
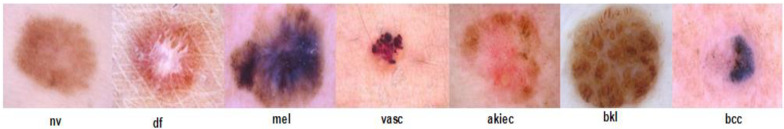
Sample images of ISIC 2018 dataset.

**ISIC 2018 Dataset**: The ISIC 2018 dataset was published by the International Skin Imaging Collaboration (ISIC) as a large-scale dataset of dermoscopy images that included over 12,500 images. The dataset performs three different tasks, i.e., lesion segmentation, attribute detection, and disease classification, respectively [34]. For the classification task, this dataset consists of more than 10,000 images of seven type of classes [35]. The sample images are illustrated in Figure 2. The ISIC 2018 challenge has two main problems: first, the limited number of images in some classes; and, secondly, the imbalanced number of images in different classes makes the classifier difficult to correct classification.

## 4. Proposed Methodology

**Figure 3:**
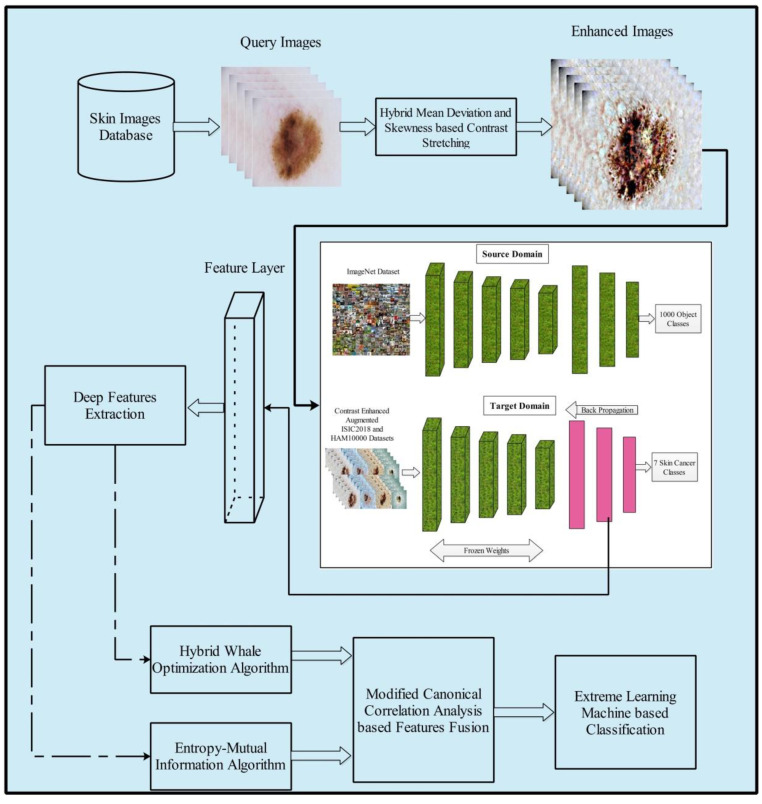
Proposed multiclass skin lesion classification using optimal deep learning features fusion.

The proposed method for multiclass skin cancer classification used deep learning and the fusion of the best selected features, and is presented in this section. The architecture of the proposed method is illustrated in Figure 3. Five primary steps are performed in the proposed method: image acquisition and contrast enhancement; deep learning features extraction using transfer learning; selection of best features using hybrid whale optimization and entropy-mutual information (EMI) approach; fusion of selected features using modified canonical correlation based approach; and, finally, ELM-based classification. The detail of each step is given below subsections.

### 4.1. Contrast Enhancement

**Figure 4:**
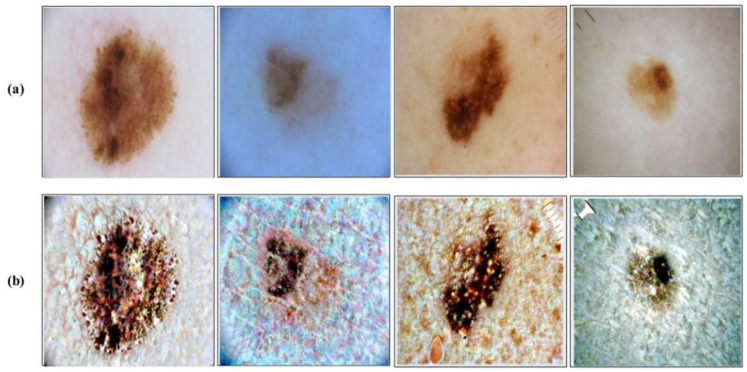
Contrast enhancement results. (**a**) Original dermoscopic images; (**b**) Proposed contrast enhancement.

Contrast enhancement is an important step in the area of medical imaging to improve the local contrast of an image. In medical image processing, contrast stretching is usually applied for accurate skin lesion detection; however, in this step, contrast stretching is applied for the sake of more useful feature extraction. Many techniques are presented in the literature for global contrast enhancement, but a very few of them focused on local contrast stretching. In this work, a hybrid contrast stretching technique is proposed based on absolute mean deviation and a skewness function. This method increases the contrast of skin lesion region and makes the image more useful for the further processing. Mathematically, this process is defined as follows:

Consider Δ is dermoscopic database, X is an input image of dimension N×M. Let, n represent the total number of pixels in the image and $x_{i}$ is the each image pixel. The absolute mean deviation (AMD) and skewness are formulated through Equations (1) and (2).

(1) $$\tilde{MD}=\frac{1}{n}\overset{n}{\underset{i=1}{\sum }}\left|x_{i}-\bar{\phi }\left(X\right)\right|$$

(2) $$\tilde{SK}=\frac{\sum_{i=1}^{n}{\left(x_{i}-\bar{X}\right)}^{3}}{\left(n-1\right)\times \sigma^{3}}$$

where the $\tilde{MD}$ is AMD of the image, $\bar{\phi }\left(X\right)$ is average mean of the dataset (Δ), and σ is the standard deviation, respectively. By employing these values, the final transformation image is obtained as follows:

(3) $$I_{1}=\left|\tilde{MD}\left(i\right)+X\right|$$

(4) $$I_{F}=\left|I_{1}-\tilde{SK}\left(i\right)\right|$$

where $I_{F}$ is final transformed image and i denote the image pixel’s. This process is applied on the entire selected datasets before training of the deep learning models. A few sample results are illustrated in Figure 4. In this figure, it is clearly illustrated that after the proposed transformation, the lesion region is more easy to identify.

### 4.2. Convolutional Neural Network

In the area of medical imaging, CNN showed promising performance for both segmentation and classification tasks. Generally, a CNN architecture consists of a number of layers, such as input layer, convolutional layer, activation layer, ReLu, pooling, fully connected, and SoftMax.

The first layer of a convolutional layer is the input layer. This layer is always in N×M×k, where k denotes the number of channels. In this layer, the whole image pixels are considered as input to the next layer. Through this layer, both low-level and high-level features are extracted.

The second layer is a convolutional layer, mainly utilized to extract feature information from the images using convolutional operation and dot product. This layer is defined as follows:

(5) $$z_{ij}^{l}=\overset{n-1}{\underset{a=0}{\sum }}\overset{n-1}{\underset{b=0}{\sum }}f_{ab}\;x_{\left(i+a\right)\left(j+b\right)}^{l-1}\;$$

where, $z_{ij}^{l}$ denotes the output of the convolutional layer. The result of this layer returned into a matrix format, which consists of the number of positive and negative values. The negative values are not required for the next step; therefore, converting those pixel values into positive ones is essential. For this purpose, an activation layer, called the ReLu layer, is applied. This layer transforms the negative pixel values into zero and keeps positive values as it is. Mathematically, it is defined as follows:

(6) f(z)= maximum(0,z)

(7) $$ReLU\left(Z\right)=\{\begin{array}{c} 0\;if\;Z<0\\ Z\;if\;Z\geq 0 \end{array}\;\;$$

**Figure 5:**
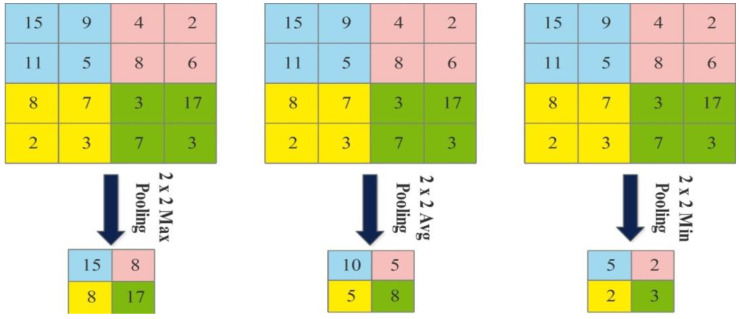
An example of max pooling, average pooling, and min pooling.

The next layer is the pooling layer, which reduces the spatial size of an image after the convolutional layer. This layer is usually applied between two convolutional layers. Visually, the pooling layer operations are illustrated in Figure 5 and mathematical output is defined as follows:

(8) $$W^{2}=\frac{W^{1}-G}{Z}+1\;$$

(9) $$H^{2}=\frac{H^{1}-G}{Z}+1\;$$

(10) $$d^{2}=d^{1}\;$$

The fully connected layer (FC) comprises neurons fully linked with all preceding layers of activations. After that, the activations can be calculated using matrix multiplication and bias offset. Finally, the performance of this layer is graded using the SoftMax classifier. Mathematically it is defined as:

(11) $$\mu \left(\vec{q}\right)i=\frac{e^{x^{i}}}{\sum_{j=1}^{n}e^{x^{j}}}\mu \left(q\right)i=\frac{e^{x^{i}}}{\sum_{j=1}^{n}e^{x^{j}}}$$

(12) $$q=\left(\begin{array}{c} \begin{array}{c} q1\\ .\\ .\\ . \end{array}\\ qn \end{array}\right)\;$$

A SoftMax function is used to take either positive, zero, or negative real value as input to all the values of $p_{i}$. As an input vector, each value is subjected to an exponential equation.

### 4.3. Deep Learning Features Extraction

**Figure 6:**
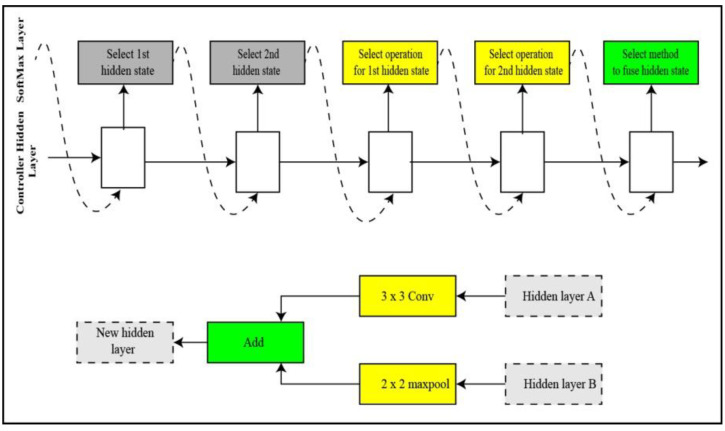
Illustration of controller process for NasNet large.

**NASNET Large:** A neural architecture search (NAS) [36] deep network trained over a million images of the challenging image database ImageNet [37]. The input size of image for the NasNet Large is 331-by-331. A child network of unique structure is created in NAS by the recurrent neural network (RNN). The child networks are trained using a holdout method to achieve accuracy. The controller is updated using the combined accuracy of child networks to create a better architecture for the network. The controller structure is represented in Figure 6. The controller predictions are gathered into A blocks. Five unique SoftMax classifiers make five predictions at five steps of the block in accordance with the discrete choices of blocks.

**Figure 7:**
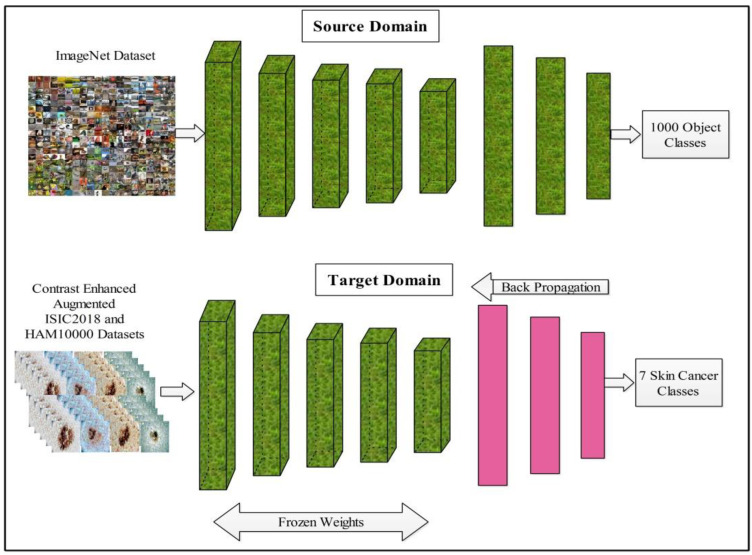
Transfer learning based model learning and features extraction.

In the process of model fine-tuning, we first remove the last three layers of this model and add a new layer according to the number of dataset classes. After the fine-tuning process, transfer learning is employed for the training of the model. In the training process, several hyperparameters are selected, such as the learning rate of 0.001, epochs of 100, minimum batch size of 64, and SGD for learning. The transfer learning process is illustrated in Figure 7. After the training of a finely tuned model on skin datasets, features are extracted from the average pool layer and utilized for the further processing, such as feature selection.

### 4.4. Optimal Feature Selection

The selection of the best features is an important step, with the advantage of improving the classification accuracy and reducing the computational time [38]. In this work, two methods are applied for the selection of best features. In the first method, a hybrid whale optimization algorithm is proposed. HWO Algorithm: Originally, the WOA was introduced by Mirjalili [39]. This algorithm is executed in two phases: (i) encircling prey and (ii) search for prey. In other words, two steps are performed: an exploitation phase and an exploration phase. Mathematically, these phases are formulated as:

(13) $$\psi =\left|V_{1}*\tilde{B^{*}}\left(t\right)-\tilde{B}\left(t\right)\right|$$

(14) $$\tilde{B}\left(t+1\right)=\tilde{B^{*}}\left(t\right)-V_{2}.\psi$$

(15) $$V_{1}=2.r$$

(16) $$V_{2}=2\alpha .\;r-\alpha$$

where $\tilde{B}$ is position vector, $\tilde{B^{*}}$ is best solution, $V_{1}$ and $V_{2}$ are coefficient vectors, r is random vector of value [0, 1], α is decrease linearly from 2 to 0 over the iterations, and |.| is absolute value, respectively. The $\tilde{B}\left(t+1\right)$ update the positions according to the best known solutions $\tilde{B^{*}}$. The values $V_{1}$ and $V_{2}$ are located in the whale according to their best solutions. The shrinking behavior is updated through the following equation:

(17) $$\alpha =2-t\frac{2}{maxIt}$$

Later, the distance is computed among $\tilde{B}$ and $\tilde{B^{*}}$ to create the position of a neighbor search agent.

(18) $$\tilde{B}\left(t+1\right)=\psi^{\prime }.\;e^{bh}.\cos\left(2\pi h\right)+\tilde{B^{*}}\left(t\right)$$

(19) $$\psi^{\prime }=\left|\tilde{B^{*}}\left(t\right)-\tilde{B}\left(t\right)\right|$$

where b is a constant and value is 1, and h is a random number between −1 and 1. The final shrinking process is formulated through the following equation:

(20) $$\tilde{B}\left(t+1\right)=\{\begin{array}{c} \tilde{B}\left(t+1\right)\leftarrow \mathrm{Equation}\;(14)\;if\;(ran<0.5)\;\\ \tilde{B}\left(t+1\right)\leftarrow \mathrm{Equation}\;(18)\;if\;\left(ran\geq 0.5\right) \end{array}$$

where the ran is a random number of value between 0 and 1. In the exploration phase, a random search agent is selected to guide the search.

(21) $$\psi =\left|V_{1}.{\tilde{B}}_{rand}-\tilde{B}\right|$$

(22) $$\tilde{B}\left(t+1\right)=\left|{\tilde{B}}_{rand}-\psi .\;V_{2}\right|$$

where ψ is a random search agent and $\tilde{B}\left(t+1\right)$ is selected agents (features). The extreme learning machine (ELM) classifier [40] is adopted for the classification error and to make the balance among classification classes, the following fitness function is employed:

(23) $$Fit=\alpha \varphi_{r}\left(\psi \right)+\beta \frac{\left|R\right|}{\left|Total\;Features\right|}$$

where $\varphi_{r}$ is classification error computed from ELM. The best solutions are picked and saved in $\tilde{B^{*}}\left(t\right)$. These best solutions are further refined using the sorting and absolute mean deviation (AMD)-based function. AMD is computed through Equation (1) and final selection function is defined as follows:

(24) $$Ftn=\{\begin{array}{c} \hat{\tilde{B^{*}}\left(t\right)}\;for\;\tilde{B^{*}}\left(t\right)\geq \tilde{MD}\\ Discard,\;Elsewhere \end{array}$$

EMI Selection Technique: Another technique, named Fuzzy Entropy Mutual Information (EMI), is proposed for features uncertainty handling. The fuzzy entropy is initially computed from the original feature vector and then embedded into the mutual information equation for the final selection. Fuzzy entropy is defined as follows:

(25) $$Fuzzy\;\left(E\right)=-K\overset{n}{\underset{i=1}{\sum }}\left\{{\bar{x}}_{i}\log\left({\bar{x}}_{i}\right)+\left(1-{\bar{x}}_{i}\right)\log\;p\left(1-{\bar{x}}_{i}\right)\right\}$$

Using this equation, the join entropy of two variables is computed as follows:

(26) $$J\left(E\right)=\underset{x_{i},x_{j}}{\sum }p\left(x_{i},\;x_{j}\right)\log\left(x_{i},\;x_{j}\right)$$

(27) $$J\left(x_{i}|x_{j}\right)=-\underset{x_{i},\;x_{j}}{\sum }p\left(x_{i},\;x_{j}\right)\log\;p\left(x_{i}|x_{j}\right)$$

After that, the common information is computed among $x_{i}$ and $x_{j}$, known as mutual information.

(28) $$I\left(x_{i};x_{j}\right)=-\underset{x_{i},\;x_{j}}{\sum }p\left(x_{i},\;x_{j}\right)\log\left(\frac{p\left(x_{i},\;x_{j}\right)}{p\left(x_{i}\right),\;p\left(x_{j}\right)}\right)$$

The resultant vector $I\left(x_{i};x_{j}\right)$ is finally fused with the HWOA-based selected feature vector using modified canonical correlation analysis (MdCAA).

### 4.5. Optimal Feature Fusion

The best selected features of HWO and EMI are finally fused in one matrix using modified canonical correlation analysis. Mathematically, the original CCA is formulated as follows:

(29) $${\left\{x_{i}\right\}}_{i=1}^{n}\;\varepsilon \;S^{j}\;,\;{\left\{y_{j}\right\}}_{j=1\;}^{n}\varepsilon \;S^{k}\;,\;{\left\{z_{k}\right\}}_{k=1}^{n}\;\varepsilon \;S^{l}$$

where *j*, *k*, and *l* represent dimensions of sample space, n denotes observation size. CCA aims to find projection directories.

(30) $$a_{x}\;\varepsilon \;S^{j}\;,\;a_{y}\;\varepsilon \;S^{k},a_{z}\;\varepsilon \;S^{l}\;$$

That raises the correlation between $a_{x}^{T}X,\;a_{y}^{T}Y,a_{z}^{T}Z\;$ where *X* = [$x_{1},\;x_{2},\ldots x_{n}]\;$, *Y* = [$y_{1},\;y_{2},\ldots y_{n}]$ and *Z* = [$z_{1},\;z_{2},\ldots z_{n}]$ represents sample matrices. Formally, in CCA we solve:

(31) $$\rho =max\;\frac{a_{x}^{T}a_{y}\;Z_{xyz\;}a_{z}}{\sqrt{(a_{x}^{T}}\;Z_{xx\;}a_{x})\;(a_{y\;}^{T}Z_{yy\;}a_{y})\;(a_{z\;}^{T}Z_{zz\;}a_{z})\;}\;$$

where $Z_{xyz\;}=XYZ^{T}$ define covariance matrix between feature sets and $Z_{xx}=XX^{T}$, $Z_{yy}=YY^{T}$, $Z_{zz}=ZZ^{T}$ represents covariance within three feature sets. When the matrices within feature sets are non-singular, then CCA can be obtained by computing generalized Eigen-problem.

(32) $$\left[\begin{array}{ccc} Z_{xyz\;}\;Z_{zz}^{-1}\;Z_{zxy\;} & 0 & 0\\ 0 & Z_{zxy\;}\;Z_{yy}^{-1}\;Z_{yzx\;} & 0\\ 0 & 0 & Z_{yxz\;}\;Z_{xx}^{-1}\;Z_{xzy\;} \end{array}\right]\left[\;\begin{array}{c} A_{x}\\ A_{y}\\ A_{z} \end{array}\right]=\lambda \;\left[\begin{array}{c} A_{x}\\ A_{y}\\ A_{z} \end{array}\right]$$

Let $A_{x}$ = $\left[a_{x1},\;a_{x2},\ldots a_{xn}\right]$, $A_{y}$ = $\left[a_{y1},\;a_{y2},\ldots a_{yn}\right]$, $A_{z}$ = $\left[a_{z1},\;a_{z2},\ldots a_{zn}\right]$ denote three projection directories matrices. Where the vector pairs ${\left(a_{xi},a_{yi},\;a_{zi}\right)}_{i=1}^{d}$ corresponds to the largest d generalized Eigen value. From the three modalities we can get the fused feature as termed below:

(33) $$F\;\left(i\right)=\left[\;\begin{array}{c} A_{x}^{T\;}x\;\\ A_{y}^{T}\;y\\ A_{z}^{T}\;z \end{array}\right]$$

where F (i) is the fused vector that later sorted into descending order and remove the redundant features by comparing approach. The final results features are finally classified using ELM classifier.

### 4.6. Extreme Learning Machine

**Figure 8:**
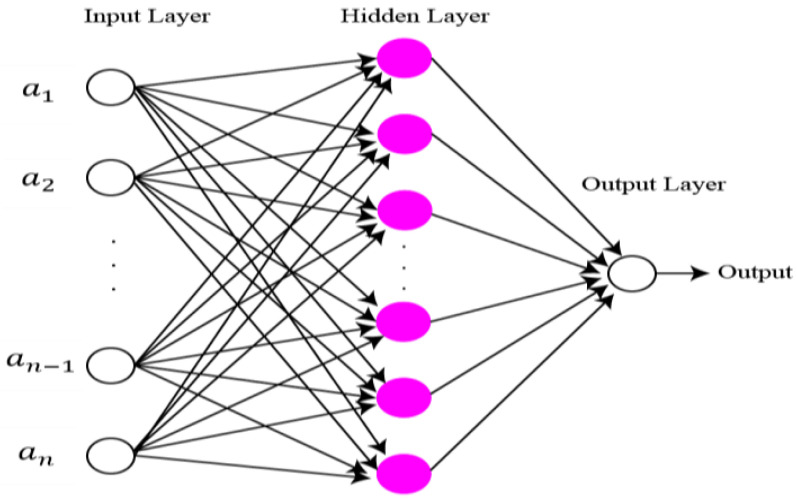
Structure of ELM classifier.

The final features are classified using extreme learning machine (ELM) [41]. The structure of ELM is illustrated in Figure 8. Compared to classical gradient-based neural networks (GBNN), the ELM rate of learning and generalization efficiency is better. In the case of the ELM algorithm, the assignment of weights and hidden biases are carried out randomly. Although a least square technique is used for the calculation of output weight. Mathematical representation of ELM is as follows:

Suppose ELM has S unique samples $\left(a_{i},b_{i}\right)$ from the fused feature set. The approximation of the desired output can be carried out with help of ELM having zero error which can be denoted as

(34) $$b_{i=}|\sum_{j=1}^{L}\beta_{j}g\left(\omega_{j}.a_{i}+c_{j}\right),i=1,\ldots ,S$$

where $a_{i}$ is input samples and $b_{i}$ is output samples. L refers to nodes of hidden layer, $c_{i}$ are weights for output, activation function is referred to as g(.). $\varepsilon_{j}$ and $c_{j}$ refer to input weights and input bias, respectively. The activation function for ELM is the radial basis function. The matrix of Equation (35) can be denoted as:

(35) B=Hβ 

where the weights of matrix are represented as $\beta =\left[\beta_{1},\beta_{2},\ldots ,\;\beta_{L}\right]$, the target output is represented as $B=\left[b_{1},b_{2},\ldots ,\;b_{N}\right]$, the hidden layer output can be represented as H.

(36) $$H=\left[\begin{array}{ccc} g\left(\varepsilon_{1}.a_{1}+c_{1}\right) & \cdots & g\left(\varepsilon_{L}.a_{1}+c_{L}\right)\\ \vdots & \ddots & \vdots \\ g\left(\varepsilon_{1}.a_{N}+c_{1}\right) & \cdots & g\left(\varepsilon_{L}.a_{N}+c_{L}\right) \end{array}\right]$$

Practically, the nodes of hidden layer (L) are less than the total number of samples of training (*S*). The matrix c of the output weight is not a singular matrix. The ELM approximation for target output cannot be with zero error. That is why the Moore Penrose (MP) generalization inverted method is utilized to obtain approximation and to express output weights.

(37) $$\beta^{*}=H^{‡}$$

where $H^{‡}$ refers the generalized inverse of hidden layer of output.

## 5. Results and Analysis

The proposed multiclass skin lesion classification framework results are presented in this section. Two publicly available datasets, named HAM10000 [32] and ISIC2018 [42], are employed for the experimental process. Both datasets consist of seven different skin lesion classes. As mentioned in Section 4.1, the data augmentation process is employed in this work to handle the imbalanced issue. Therefore, the balanced datasets are considered for the experimentations. Several parameters are employed in this work during the training process, such as a training rate of 0.001; a mini batch size of 64, and max epochs of 500. The 50:50 is considered for the training and testing process for both datasets, whereas the cross validation value is 10. Several classifiers are utilized for the experimental process, such as multiclass SVM (MC-SVM), fine KNN (F-KNN), decision trees (DT), Naïve Bayes, ensemble tree (EBT), and single hidden layer extreme learning machine (ELM). The performance of each classifier is computed using accuracy, precision, FDR, and computational time. The simulations of proposed framework are performed on MATLAB2020b. Moreover, an 8GB graphics processing unit (GPU) is utilized for processing of the proposed framework.

**Experiments:** The following experiments are performed in this work for validation of the proposed framework:

- Classification using originally deep features;
- Classification using HWOA-based best features selection;
- Classification using EMI-based best features selection;
- Classification using best selected features fusion approach.

### 5.1. Results on HAM10000 Dataset

Table 1 presents the classification results of HAM10000 dataset using originally deep extracted features from fine-tuned NasNet large network. The features are extracted from the global average pool layer and the classification is performed. On this layer, the length of extracted deep features is N×1056. The highest achieved accuracy noted in this table is 84.90%, for the ELM classifier. The precision and FDR values are 84.10 and 15.90%, respectively. Additionally, the computational time of ELM is 214.5536 (s). A few other classifiers are also implemented, such as MC-SVM, F-KNN, DT, Naïve Bayes, and EBT, and the accuracy value is 84.72, 80.90, 79.63, 81.50, and 82.96%, respectively. The accuracy and precision value of ELM is better, compared to these classifiers. Additionally, computationally, ELM is more efficient than rest of the classifiers.

After the experimentation on the originally deep features, two feature selection techniques are introduced, named HWOA and EMI. Table 2 presents the classification results of the HWOA-based best features selection algorithm. The higher noted accuracy in this table is 85.10%. The precision and FDR values of this classifier are 84.86 and 15.14%, respectively. Computation time of ELM is 147.6329 (s), which is less as compared to the other mentioned classifiers, such as MC-SVM, F-KNN, to name a few. Compared to the results of HWOA-based feature selection with originally deep extracted (from Table 1), it is noted that a minor improvement is occurred in the accuracy but there is significant change in the computational time.

Table 3 presents the classification accuracy using EMI-based best feature selection approach. In this table, the best noted accuracy value is 84.90, whereas the computational time is 90.3560 (s). This selection method has also shown slight improvement in the accuracy but there is a high change in the computational time (compared with Table 1 and Table 2). Based on these three experiments, it is analyzed that the selection of the best features shows slight improvement in the accuracy value but higher change in the computational time. After the process of best features selection, time is significantly reduced.

**Figure 9:**
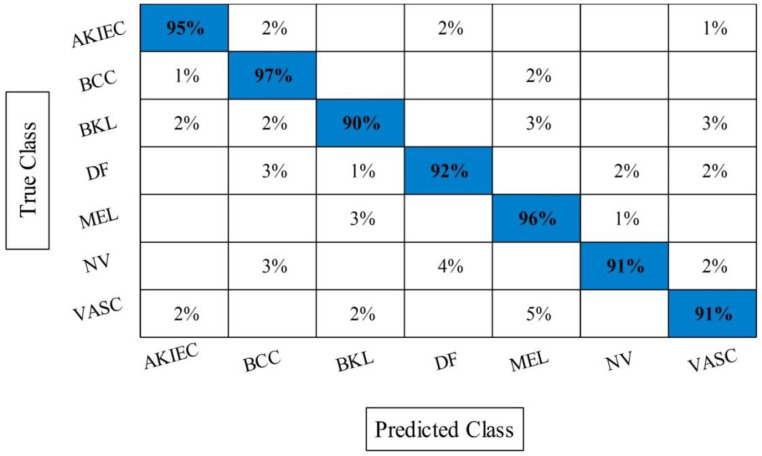
Confusion matrix of ELM after best selected features fusion for HAM10000.

**Figure 10:**
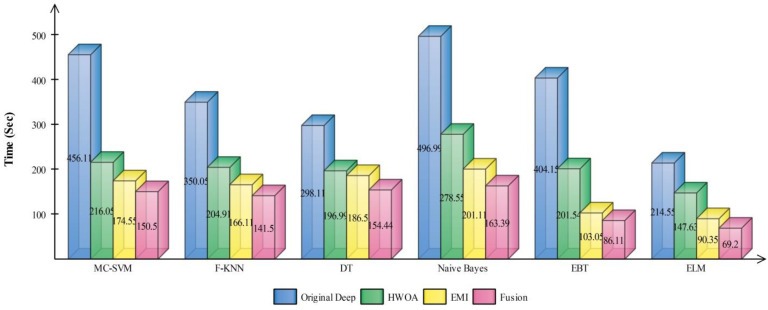
Computational time based comparison among middle steps for HAM10000.

The best selected features through HWOA and EMI are finally fused using proposed fusion approach. Table 4 presents the results of features fusion. In this table, the best noted accuracy value is 93.40% that is improved than the accuracy values given in Table 1, Table 2 and Table 3. After the fusion process, the change in the accuracy value is approximately 8%. The values of precision and FDR is 93.10 and 6.90%, respectively. Additionally, the computational time is reduced and reached to 69.2036 (s). The recent best time was 90.3560 (s) for EMI-based features selection approach. For the rest of the classifiers, such as MC-SVM, F-KNN, DT, Naïve Bayes, and Ensemble trees, also showed the improved performance after the fusion process. The accuracy value of ELM is further verified by Figure 9 in the form of confusion matrix. In this figure, the diagonal value represents the correct prediction rate. Figure 10 illustrated the overall depiction of computational time for all four experiments using HAM10000 dataset. Based on this figure, it is clearly analyzing that the fusion process is computationally less expensive than original deep features, HWOA, and EMI. The fusion process reduced some features that are not meet the criteria, given in Equation (33). Moreover, the ELM is performed overall better than rest of the classifiers.

### 5.2. Results on ISIC2018 Dataset

The classification results on ISIC2018 dataset are presented in this section. Several experiments are performed to validate the propose results. Table 5 presents the classification results of ISIC2018 dataset using originally deep extracted features. The ELM classifier achieved a highest accuracy of 85.96%, whereas the precision value is 85.52%. The rest of the classifiers achieved an accuracy of 83.90, 81.40, 82.56, 83.50, and 82.16%, respectively. The computational time of each classifier is also noted and minimum noted time is 286.1009 (s). To overcome the problem of higher computational time and improved accuracy, a hybrid WOA is proposed. The results of HWOA are given in Table 6. This table presents the best accuracy of 87.86%, whereas the computational time is 184.5294 (s). The computed accuracies of other classifiers are 85.14, 82.96, 84.60, 85.36, and 83.90%, respectively. This results in this table showing that the use of HWOA improved in the accuracy and minimizes the computational time. To further reduce the computational time, another feature selection technique is proposed named EMI. The results of this approach are given in Table 7. This table showed that the accuracy is almost consistent but the computational time is minimized.

**Figure 11:**
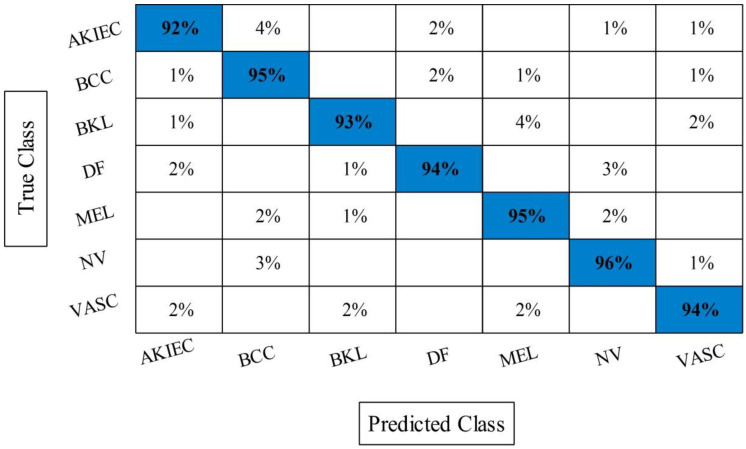
Confusion matrix of ELM after best selected features fusion for ISIC2018 dataset.

**Figure 12:**
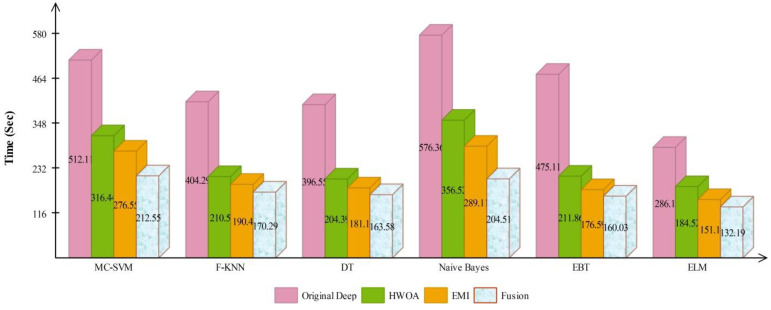
Computational time based comparison among middle steps for ISIC2018 dataset.

The accuracy of HWOA- and EMI-based feature selection is not improved as compared to the existing techniques; therefore, a fusion technique is proposed. Table 8 presents the results of features fusion. In this table, the best noted accuracy value is 94.36% that is improved than the accuracy values given in Table 5, Table 6 and Table 7. After the fusion process, the change in the accuracy value is approximately 7%. The computational time is also reduced after the fusion process and reached to 132.1990 (s). The recent best time was 184.5294 (s) for EMI-based features selection approach. For the rest of the classifiers accuracy is also improved (91.84, 90.60, 92.56, 92.80, and 90.46%, respectively). Figure 11 illustrated the confusion matrix of ELM classifier for ISIC2018 dataset. Through this figure, the ELM accuracy can be verified. Figure 12 illustrated the overall depiction of computational time for all four experiments using ISIC2018 dataset. Based on this figure, it is clearly analyzed that the fusion process is computationally less expensive. Moreover, ELM classifier is computationally less expensive than other listed classifiers in this table.

### 5.3. Discussion

**Figure 13:**
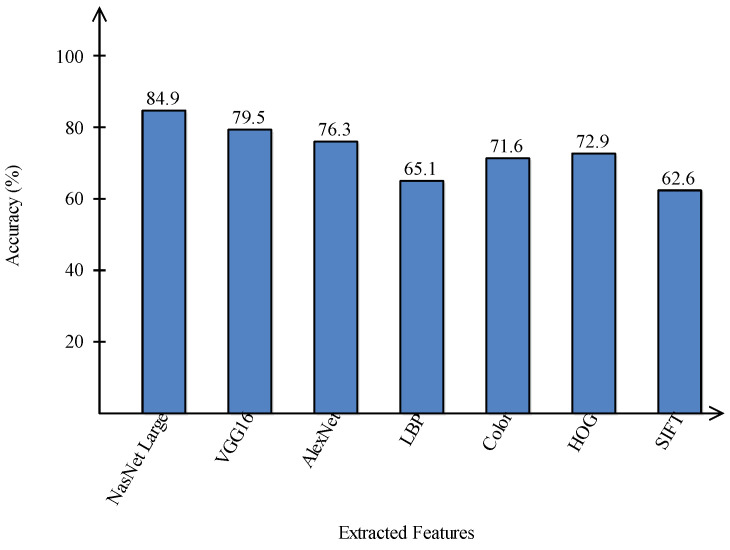
Comparison of deep learning and traditional features in terms of accuracy on HAM10000 dataset.

**Figure 14:**
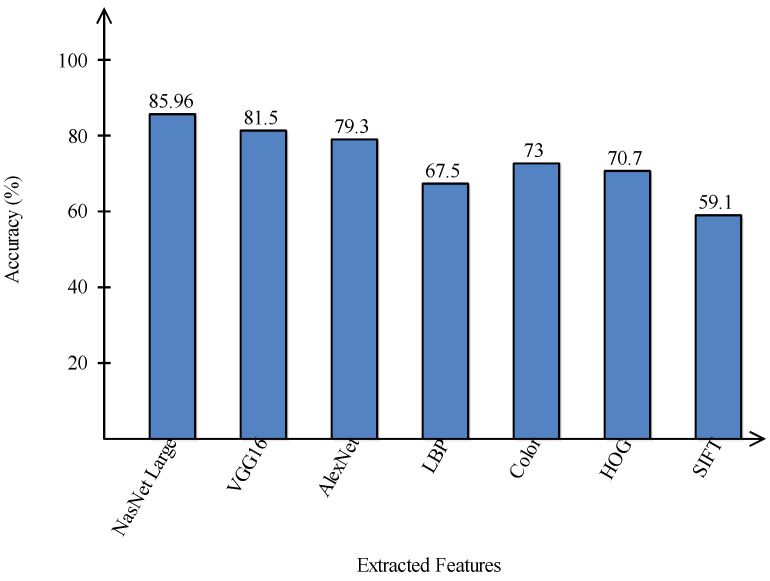
Comparison of deep learning and traditional features in terms of accuracy on ISIC2018 dataset.

**Figure 15:**
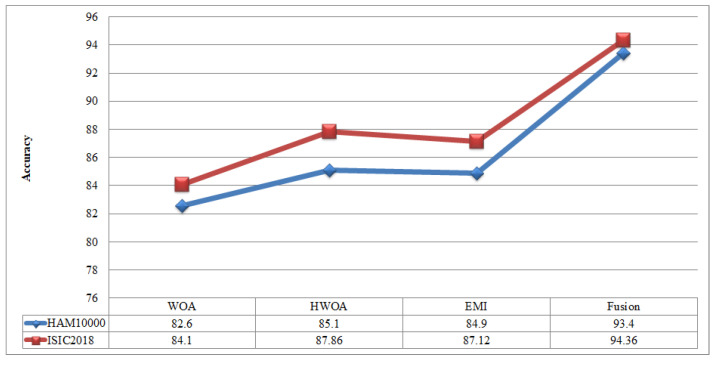
Comparison among original WOA with HWOA, EMI, and Fusion techniques.

A comparison is conducted among extracted deep features and traditional features, as illustrated in Figure 13 and Figure 14. In these figures, it is illustrated that the deep learning-based extracted features (i.e., NAsNet Large, VGG16, Alexnet) give better results than traditional features (i.e., HOG, LBP, SIFT, and Color) on selected datasets. In the traditional features, HOG and color give better results than SIFT and LBP. In addition, Figure 15 showing the comparison among original WOA-, HWOA-, and EMI-based best feature selection.

**Figure 16:**
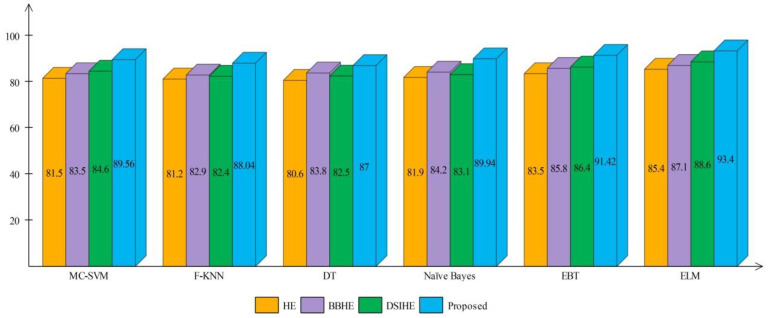
Comparison of different contrast enhancement techniques for HAM10000 dataset.

**Figure 17:**
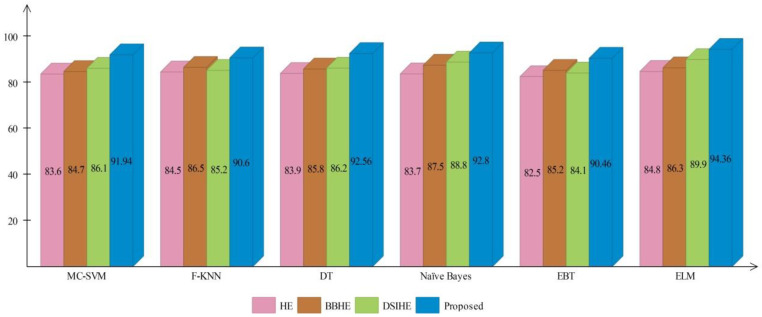
Comparison of different contrast enhancement techniques for ISIC2018 dataset.

A comprehensive analysis of different contrast enhancement techniques is also performed, with the proposed enhancement technique as the chosen enhancement technique. In place of the proposed enhancement technique, three well-known techniques, such as HE [43], BBHE [44], and DSIHE [45], are chosen and included in the proposed framework. Figure 16 and Figure 17 show the computed and plotted results after the addition of these techniques. Based on these figures, it is clear that the proposed contrast enhancement technique produces superior results. The second best accuracy is provided by the DSIHE contrast enhancement technique.

Finally, a comparison is also conducted with state of the art (SOTA) techniques, as shown in Table 9. In this table, Huang et al. [46] used the HAM10000 dataset for the experimental process and achieved an accuracy of 85.80%. Hamsi et al. [47] achieved an accuracy of 87.70% for the HAM10000 dataset. Chaturvedi et al. [48] improved accuracy on Ham10000 and reached to 92.83%. Recently, Khan et al. [26] introduced a deep learning framework and obtained an accuracy of 86.50% on the Imbalanced HAM10000 dataset. Authors in [49,50] introduced a deep learning-based framework and achieved an accuracy of 92.40% and 93.4%. In the proposed method, the obtained accuracy is 93.40% on HAM10000 dataset and 94.36% on ISIC2018 dataset. This showed that the proposed method outperformed than SOTA.

The key strength of this work is the selection of optimal features. The fusion process of optimal features shows better results. The key limitation of this work is high computational time due to more number of steps. In the future, the lesion segmentation step will be adopted and utilized for the direct deep learning features extraction process. Moreover, the ISIC2019 and ISIC2020 datasets will be employed for the experimental process.

## 6. Conclusions

This paper proposes an automated deep learning-based framework for multiclass skin lesion classification. The proposed method includes a number of important steps, including contrast enhancement of the skin lesion using a new function, deep learning model learning via TL using enhanced images, best feature selection via two techniques—HWO and EMI, fusion of selected features via a modified CCA approach, and finally ELM-based classification. The experimental process demonstrates that the proposed method outperformed the other methods on the chosen datasets. Overall, it is concluded that:

- The contrast enhancement step improve the quality of lesion region that later extracts the more relevant features;
- The selection of optimal features improves the classification accuracy and reduces the computational time;
- The fusion process further helps in the improvement of classification accuracy and reduces some redundant features through comparing approach.

In the future, the recent deep learning models shall be considers and refine in the middle layers [51,52,53]. Augmentation of datasets is important step and it can be refining through more up-to-date methods [54,55]. Moreover, the optimization through some latest techniques should be opted [56,57]. 

## Figures and Tables

**Table 1:** Classification accuracy using originally extracted deep features for HAM10000.

Classifiers	Performance Measures			
	Accuracy (%)	Precision (%)	FDR (%)	Time (s)
MC-SVM	84.72	84.02	15.98	456.1129
F-KNN	80.90	80.20	19.80	350.0492
DT	79.63	78.94	21.06	298.1104
Naïve Bayes	81.50	80.76	19.24	496.9860
EBT	82.96	82.16	17.84	404.1542
**ELM**	**84.90**	**84.10**	**15.90**	**214.5536**

**Table 2:** Classification accuracy using HWOA based feature selection for HAM10000 dataset.

Classifiers	Performance Measures			
	Accuracy (%)	Precision (%)	FDR (%)	Time (s)
MC-SVM	85.13	84.76	15.24	216.0492
F-KNN	81.06	80.60	19.40	204.9106
DT	79.94	79.62	20.38	196.9946
Naïve Bayes	82.69	82.13	17.87	278.5543
EBT	83.58	82.96	17.04	201.5436
**ELM**	**85.10**	**84.86**	**15.14**	**147.6329**

**Table 3:** Classification accuracy using EMI-based feature selection for HAM10000 dataset.

Classifiers	Performance Measures			
	Accuracy (%)	Precision (%)	FDR (%)	Time (s)
MC-SVM	85.00	84.62	15.38	174.5506
F-KNN	80.96	80.46	19.54	166.1056
DT	80.20	80.02	19.98	186.5092
Naïve Bayes	82.13	81.98	18.02	201.1136
EBT	83.86	83.40	16.60	103.0542
**ELM**	**84.90**	**84.74**	**15.26**	**90.3560**

**Table 4:** Classification accuracy using selected features fusion approach for HAM10000 dataset.

Classifiers	Performance Measures			
	Accuracy (%)	Precision (%)	FDR (%)	Time (s)
MC-SVM	89.56	89.14	10.86	150.5042
F-KNN	88.04	87.68	12.32	141.5036
DT	87.00	86.92	13.08	154.4432
Naïve Bayes	89.94	89.54	10.46	163.3940
EBT	91.42	91.10	8.90	86.1152
**ELM**	**93.40**	**93.10**	**6.90**	**69.2036**

**Table 5:** Classification accuracy using originally extracted deep features for ISIC2018 dataset.

Classifiers	Performance Measures			
	Accuracy (%)	Precision (%)	FDR (%)	Time (s)
MC-SVM	83.90	82.98	17.02	512.1129
F-KNN	81.40	81.16	18.84	404.2904
DT	82.56	81.86	18.14	396.5526
Naïve Bayes	83.50	82.90	17.10	576.3625
EBT	82.16	81.78	18.22	475.1108
**ELM**	**85.96**	**85.52**	**14.48**	**286.1009**

**Table 6:** Classification accuracy using HWOA-based feature selection for ISIC2018 dataset.

Classifiers	Performance Measures			
	Accuracy (%)	Precision (%)	FDR (%)	Time (s)
MC-SVM	85.14	84.76	15.24	316.4428
F-KNN	82.96	82.12	17.88	210.5006
DT	84.60	84.06	15.94	204.3992
Naïve Bayes	85.36	84.90	15.10	356.5216
EBT	83.90	83.42	16.58	211.8692
**ELM**	**87.86**	**87.40**	**12.60**	**184.5294**

**Table 7:** Classification accuracy using EMI-based feature selection for ISIC2018 dataset.

Classifiers	Performance Measures			
	Accuracy (%)	Precision (%)	FDR (%)	Time (s)
MC-SVM	84.29	83.80	16.20	276.5540
F-KNN	82.60	82.14	17.86	190.4029
DT	84.96	84.30	15.70	181.1056
Naïve Bayes	85.70	85.28	14.72	289.1198
EBT	82.86	82.50	17.50	176.5920
**ELM**	**87.12**	**86.92**	**13.08**	**151.1036**

**Table 8:** Classification accuracy using selected features fusion approach for ISIC2018 dataset.

Classifiers	Performance Measures			
	Accuracy (%)	Precision (%)	FDR (%)	Time (s)
MC-SVM	91.84	91.52	8.48	212.5546
F-KNN	90.60	89.90	10.10	170.2918
DT	92.56	92.16	7.84	163.5829
Naïve Bayes	92.80	92.40	7.60	204.5116
EBT	90.46	90.12	9.88	160.0342
**ELM**	**94.36**	**94.08**	**5.92**	**132.1990**

**Table 9:** Comparison of the proposed method accuracy with SOTA techniques.

Methods	Year	Datasets	Accuracy (%)
[46]	2020	HAM10000	85.80
[47]	2020	HAM10000	87.70
[48]	2020	HAM10000	92.83
[26]	2021	HAM10000	86.50
[49]	2020	ISIC2018	92.40
[50]	2021	ISIC2018	93.4
**Proposed**		HAM10000	**93.40**
		ISIC2018	**94.36**

## Data Availability

Not applicable.
